# A national survey of primary care physicians’ self-reported use of and attitudes to telemedicine consultations with patients in Sweden

**DOI:** 10.1177/20552076261452903

**Published:** 2026-05-19

**Authors:** Emma Brulin, Per Nilsen, Ida Seing, Hanna Fernemark, Elin Karlsson, Janna Skagerström, Nadine Karlsson

**Affiliations:** 1 Unit of Occupational Medicine, Institute of Environmental Medicine, 27106 Karolinska Institutet, Stockholm, Sweden; 2 Division of Health and Society, Department of Health, Medicine and Caring Sciences, 4566 Linköping University, Linköping, Sweden; 3 School of Health and Welfare, 3694 Halmstad University, Halmstad, Sweden; 4 Department of Behavioral Science and Learning, 4566 Linköping University, Linköping, Sweden; 5 Regional Executive Office, Public Health Unit, 4564 Region Östergötland, Linköping, Sweden; 6 Research and Development Unit in Region Östergötland, Linköping, Sweden

**Keywords:** primary care, telemedicne consultation, national representative, Sweden, survey, physicians

## Abstract

**Objective:**

Using a nationally representative sample of primary care physicians the aim was to investigate (1) potential demographic variations (age, gender, employer, country of education and clinical experience) in self-reported use of and attitudes to telemedicine consultations and (2) whether general health, turnover intention and job satisfaction were associated with an increased self-reported use of and positive attitudes toward telemedicine consultations.

**Methods:**

This cross-sectional study utilised data from the 2022 Longitudinal Occupational Health survey in Healthcare Sweden (LOHHCS), which included questions on medical background, work environment, and demographics, as well as use and attitude toward telemedicine consultations. Data were analysed using logistic regressions.

**Results:**

Of 7908 invited physicians, 2712 responded (34% response rate), including 1099 primary care physicians. Approximately 70% of respondents reported using telemedicine consultations, and ∼60% of those users reported very or quite positive attitudes toward telemedicine. Reported telemedicine use was strongly associated with positive attitudes. Physicians with longer clinical experience were more likely to report using telemedicine but less likely to express positive attitudes. Use of and positive attitudes towards telemedicine consultations were associated with self-reported high job satisfaction, but also high turnover intention.

**Conclusions:**

This study highlights the importance of not viewing digitalisation as a straightforward solution to job satisfaction or retention issues. While causality cannot be determined, the findings suggest that using telemedicine helps physicians appreciate its benefits, fostering positive attitudes and increasing their satisfaction with work.

## Introduction

Telemedicine consultations, also known as telehealth or e-consultations, involve healthcare providers communicating with patients remotely using digital technologies, such as mobile apps or video calls. This marks a significant change in the delivery of primary care.^1^ The number of digital consultations, distinct from traditional telephone consultations, with primary care physicians has increased considerably in Sweden over the past decade.^2^ Private healthcare companies have spearheaded the development; however, this type of service has also been adopted more recently by the 21 publicly funded regions responsible for providing healthcare in Sweden.^2^ In 2019, approximately 1 million digital consultations with physicians took place in Sweden,^2^ while the majority of primary care physicians reported not using telemedicine, including digital consultations, chat, and emails.^3^ The COVID-19 pandemic led to a rapid expansion of telemedicine use, as new methods were needed to keep patients connected to healthcare when social distancing and other measures restricted mobility and access.^4–7^ The number of digital consultations more than doubled to 2.3 million in 2020, representing 18% of the “physical” consultations with primary care physicians.^2^

Research has shown that telemedicine consultations can be cost-effective and provide advantages for patients, providers, and taxpayers.^8–12^ Advantages for patients include greater access to healthcare, particularly for individuals in more remote areas, and reduced exposure to viruses and other pathogens.^8,11^ Monitoring chronic diseases through telemedicine has demonstrated evidence of improved health outcomes comparable to those in traditional healthcare.^10^ Furthermore, telemedicine facilitates focused discussions and continuity of care, particularly during periods of social distancing.^13^ Meanwhile, from a system perspective, there is a risk that telemedicine will shift resources from those with higher healthcare needs to those with lower needs.^11,14^ This is emphasised by the fact that telemedicine users in Sweden tend to be younger, more likely to reside in urban areas, to have favourable socioeconomic conditions, and to have higher levels of education than the general population and users of traditional primary care.^2^ Furthermore, women have been shown to use telemedicine more frequently than men.^15^ Concern has been raised that the competence of primary care physicians could be compromised if telemedicine consultations predominantly focus on less complex medical problems.^12^

Existing studies indicate that, from physicians’ perspectives, telemedicine may positively affect job satisfaction and workload^16^ and reduce burnout.^17^ On the other hand, introducing telemedicine may also increase physicians’ workload as they need to learn new tasks and navigate technical barriers, including insufficient infrastructure.^18,19^ Much of the existing research has primarily focused on patients’ perspectives and their experiences with digital consultations,^16,18,20–22^ as well as technical issues such as usability, cost-effectiveness, platforms used, user characteristics, application challenges, and facilitators.^16^ Only a few small studies have investigated the extent to which Swedish primary care physicians use telemedicine consultations with patients or their attitudes toward telemedicine.^3,12,23^ Against this backdrop, the purpose of this study was to investigate, using data from a nationally representative survey, the self-reported use and attitudes of primary care physicians in Sweden toward telemedicine consultations (via digital technology) with patients. The specific aims were to investigate (1) potential demographic variations (age, gender, employer, country of education, and clinical experience) in self-reported use of and attitudes toward telemedicine consultations and (2) whether general health, turnover intention, and job satisfaction were associated with self-reported use of and positive attitudes toward telemedicine consultations.

## Methods

### Study setting

The survey was conducted in Sweden, a country in northern Europe with about 11 million inhabitants. Healthcare in Sweden is the responsibility of 21 regions (formerly known as county councils). All citizens are insured by the state to ensure equal access to healthcare, and fees are standardised and regulated by law.^24^ The physician/patient ratio is 4.3/1000, and the nurse & midwife/patient ratio is 11.4/1000.^25^ As the first line of care in Sweden, primary care is tasked with treating patients without limitations on disease type, age, or specific patient groups.^26^ Primary care is responsible for medical judgment, treatment, and preventive work, as well as rehabilitation that does not require specialised care.^26^

Swedish primary care consists of public actors and two types of private actors: those with region contracts to provide healthcare at the same out-of-pocket cost for citizens as public healthcare, which are reimbursed from tax funding, and those without contracts, where the patients pay for the visits themselves.^27^ Almost 50% (530 of 1178 units) of all primary care units are private with public contracts.^28^

Swedish primary care physicians specialise in investigating and diagnosing a wide range of diseases and symptoms. They are also responsible for all medical concerns of the elderly. Traditionally, they conduct patient consultations via face-to-face visits, telephone calls, or home visits (e.g., rounds in a care home). Swedish primary care was significantly impacted by the pandemic,^29,30^ and physicians had to transition from face-to-face to telehealth consultations using digital tools, which is the focus of this study.

### Study design, sample, and procedure

The study had a cross-sectional design with a nationally representative sample of physicians in Swedish primary care. Data were obtained from the 2022 Longitudinal Occupational Health Survey in Healthcare Sweden (LOHHCS) study.^31^ Statistics Sweden employed a stratified random sampling method to select a representative sample of physicians from the occupational registers, based on SSYK (Standard för svensk yrkesklassificering, which translates to Standard for Swedish Occupational Classification) codes. For geographical distribution, the data were stratified by the six administrative regions. We also stratified by place of work, distinguishing between hospitals and primary care. An additional sample was drawn from those with a medical degree in the national educational register (UREG). The educational register was used because of its immediate updates, compared with the occupational register, which has a one-year delay in updates. This resulted in a representative sample of 7908 physicians in 2022, and 2712 answered the questionnaire, representing a 34% response rate. As this study focused on physicians working in primary care, we included only respondents who reported working in primary care (n = 1099) (Table 1).

**Table 1.** Characteristics of the study sample.

​	Total sample (n=1090), n (%)		Users of telemedicine consultations (*n*=721), *n* (%)		Comparison between users with positive and negative attitudes
	Not used telemedicine	Used telemedicine	Positive attitudes	Negative attitudes	
Total	322 (29.5)	768 (70.5)	431 (59.8)	290 (40.2)	​
Age (*n*=1099)					0.469
≤29 years	31 (9.6)	43 (5.6)	25 (5.8)	16 (5.5)	​
30–39 years	88 (27.3)	241 (31.4)	141 (32.7)	88 (30.3)	​
40–49 years	78 (24.2)	230 (29.9)	119 (27.6)	97 (33.4)	​
50–59 years	47 (14.6)	134 (17.4)	72 (16.7)	49 (16.9)	​
≥60 years	78 (24.2)	120 (15.6)	74 (17.2)	40 (13.8)	​
Gender (*n*=1099)					0.344
Male	130 (43.2)	319 (41.5)	172 (39.8)	126 (43.4)	​
Female	183 (56.8)	449 (58.5)	259 (60.1)	164 (56.6)	​
Employer (*n*=1098)					0.106
Region	262 (81.4)	503 (65.6)	267 (61.9)	201 (69.9)	​
Private sector	49 (15.2)	235 (30.6)	145 (33.6)	79 (27.3)	​
Other	11 (3.4)	29 (3.8)	19 (4.4)	9 (3.1)	​
Country of education (*n*=1098)					0.090
Sweden	257 (79.8)	603 (78.6)	330 (76.6)	237 (82.0)	​
Other European countries	44 (13.7)	115 (15.0)	69 (16.0)	41 (14.2)	​
Non-European countries	21 (6.5)	49 (6.4)	32 (7.4)	11 (3.8)	​
Clinical experience (*n*=1097)					0.198
<5 years	72 (22.4)	116 (15.1)	76 (17.7)	35 (12.1)	​
5–10 years	61 (19.0)	173 (22.6)	98 (22.8)	66 (22.8)	​
10–15 years	52 (16.2)	177 (23.1)	93 (21.6)	73 (25.2)	​
>15 years	136 (42.4)	301 (39.2)	163 (37.9)	116 (40.0)	​
Health (*n*=1097)					0.251
Bad	75 (23.3)	150 (19.6)	94 (21.9)	53 (18.3)	​
Good	247 (76.7)	616 (80.4)	336 (78.1)	236 (81.7)	​
Turnover intention (*n*=1098)					0.890
High	49 (15.3)	157 (20.4)	90 (20.9)	57 (19.7)	​
Low	158 (49.2)	386 (50.3)	216 (50.1)	145 (50.0)	​
None	114 (35.5)	225 (29.3)	125 (29.0)	88 (30.3)	​
Job satisfaction (*n*=1098)					0.071
Low	59 (18.3)	115 (15.0)	55 (12.8)	55 (19.0)	​
Middle	52 (16.1)	139 (18.1)	76 (17.6)	49 (17.0)	​
High	211 (65.5)	513 (66.9)	300 (69.6)	185 (64.0)	​
Use of telemedicine (*n*=1090)					<.001
Not at all	322 (100)	0 (0.0)	N/A	N/A	​
On some occasions	0 (0.0)	441 (57.4)	187 (43.4)	217 (74.8)	​
On many occasions	0 (0.0)	319 (41.5)	237 (55.0)	72 (24.8)	​
For almost all my patient consultations	0 (0.0)	8 (1.0)	7 (1.6)	1 (0.3)	​

The survey was distributed to the sample of physicians between March and May 2022. One invitation and three reminders were sent out. The respondents were informed that they could answer the questionnaire either online or by filling out the paper form sent with the second reminder. Statistics Sweden was responsible for the power calculations, sampling, distributing the questionnaires, and gathering the data. Statistics Sweden conducted an analysis of missing responses, comparing responders to the sample and the full population using population registers, and found no systematic differences. Item nonresponse in the sample ranged from 0.0% to 2.5% across the included questions. Statistics Sweden is responsible for official statistics and other government statistics and for developing and producing statistics for research and other analyses.^32^

The study followed the Strengthening the Reporting of Observational Studies in Epidemiology (STROBE) guidelines for reporting observational research.

### Questionnaire and measures

#### Telemedicine

Use of telemedicine was measured with one question: “To what extent have you used telemedicine (via digital technology) for patient consultations in the last 12 months?” The response options were as follows: “do not have patient consultations in my work”, “not at all”, “on a few occasions”, “on several occasions”, and “I have transitioned almost all patient consultations to telemedicine”. In this study, telemedicine is thus defined as the patient-healthcare provider consultations via digital technology, thereby excluding traditional telephone consultations. Use of telemedicine was dichotomised into users (those reporting usage “on several occasions” and “I have transitioned almost all patient consultations to telemedicine”), and non-users (including responses “not at all” and “on a few occasions”).

**Figure 1.**
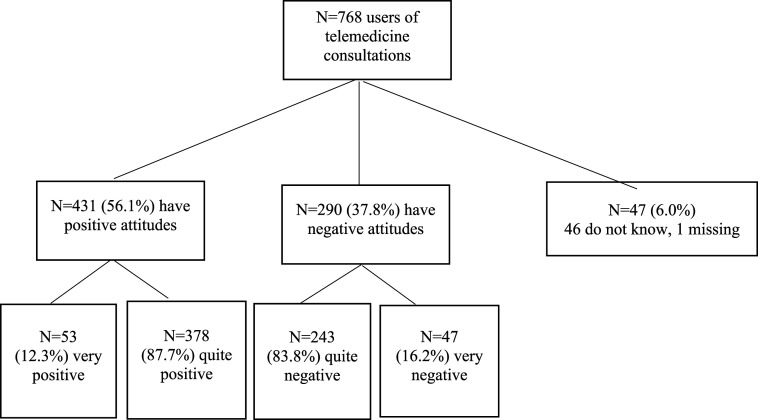
Flowchart showing distribution of attitudes towards telemedicine among users.

Attitudes to telemedicine were measured with one question: “What is your main view of using telemedicine for patient consultations?” The response options were “very positive”, “quite positive”, “quite negative”, “very negative” and “no opinion”. This question was only posed to those who reported using telemedicine in their work (n = 768). Responses from respondents who did not have an opinion regarding “What is your main view of using telemedicine for patient consultations?” were coded as missing. Attitudes towards telemedicine was dichotomised into positive attitudes (including ”very positive” and “quite positive”) and negative attitudes (including responses “quite negative” and “very negative), excluding the three respondents who did not have patient consultations in their job and those who did not use telemedicine (see Figure 1).

#### Demographic

The respondent’s age and gender were retrieved from Statistics Sweden’s registers. Age was categorised into five evenly sized groups: ≤29 years, 30–39 years, 40–49 years, 50–59 years, and ≥60 years. Gender was categorised as male or female.

The response options for the employer were: region, private employer with public funding, state, staffing agency, self-employed, or none of these (accompanied by an open-text response). The responses were merged into three categories: region, private employer (with public or private funding), and other (including state, staffing agency, self-employed, and those who answered that none of the response options applied to them).

The options for country of education were Sweden, other EU countries, Norway and other countries outside the EU. These were categorised into three groups: Sweden, other European countries, and non-European countries.

The questionnaire included four options for clinical experience: <5 years; 5–10 years; 10–15 years; >15 years.

#### General health, turnover and satisfaction

Self-rated general health was measured with a single-item question as in the Copenhagen Psychosocial Questionnaire (COPSOQ III),^33^ “In general, how do you perceive your health?” with response options on a five-point Likert scale from excellent to bad. The variable was dichotomised into bad health (including response options bad and quite good) and good health (including excellent, very good and good).

Turnover intention is an individual’s willingness to leave their current job voluntarily.^34^ Turnover intention was measured with one question: “How often during the last 12 months have you considered quitting your job?” The response options were trichotomized into high turnover intention (every day, once or a few times a week), low turnover intention (once or a few times a month and once or a few times in the last 12 months), and no turnover intention (never).

Job satisfaction refers to the extent to which a person reports overall contentment with various aspects of their work, such as career opportunities, incentives, and salary.^35,36^ Job satisfaction was measured with a single question drawn from COPSOQ III: “How satisfied or dissatisfied are you with your job?” The response options were trichotomized into three categories: satisfied (including ‘very satisfied’ and ‘quite satisfied’, coded “high job satisfaction” in tables), neither satisfied nor dissatisfied (coded “middle job satisfaction” in tables, and dissatisfied (including ‘quite dissatisfied’ and ‘very dissatisfied’, coded “low job satisfaction” in tables).

### Statistical methods

The distribution of the study sample characteristics (Table 1) was estimated for all study respondents. Descriptive statistics are presented as frequencies, accompanied by a chi-squared test to compare the characteristics of the study sample between users with positive attitudes and those with negative attitudes towards telemedicine (Table 1).

Thereafter, logistic regression was used to identify the characteristics of the study sample with respect to the two telemedicine variables (use and attitude, respectively). First, univariate associations were investigated between the outcome variables and demographics (age, gender, employer, country of education, and clinical experience; Model I in Table 2), and health, turnover intention, and job satisfaction (Model I in Table 3). In Model II, all variables were jointly added (Tables 2 and 3). For the outcome “attitudes to telemedicine”, we performed an additional model to adjust for the frequency of use of telemedicine (Model III, Tables 2 and 3). Odds ratios (ORs) were estimated with 95% confidence intervals (CIs). Results were considered statistically significant at *P*<0.05 using two-tailed tests. Statistical analyses were performed with SPSS 28.

**Table 2.** Logistic regression showing demographics of the use of and attitude toward telemedicine consultation.

​	​	Model I^a^			Model II^b^			Model III^c^		
		OR	95% CI	*P* value	OR	95% CI	*P* value	OR	95% CI	*P* Value
Use of telemedicine consultation	Age									
	≤29 years	1	​	​	1	​	​	​	​	​
	30–39 years	1.97	1.17–3.33	0.011	1.22	0.66–2.27	0.529	​	​	​
	40–49 years	2.12	1.25–3.61	0.005	1.09	0.52–2.26	0.822	​	​	​
	50–59 years	2.06	1.16–3.63	0.013	0.99	0.43–2.25	0.974	​	​	​
	≥60 years	1.11	0.65–1.91	0.708	0.54	0.23–1.29	0.167	​	​	​
	Gender									
	Male	1	​	​	1	​	​	​	​	​
	Female	1.07	0.82–1.39	0.619	0.94	0.71–1.24	0.665	​	​	​
	Employer									
	Region	1	​	​	1	​	​	​	​	​
	Private sector	2.50	1.77–3.52	<0.001	2.29	1.61–3.25	<0.001	​	​	​
	Other	1.37	0.68–2.79	0.381	1.48	0.71–3.08	0.298	​	​	​
	Country of education									
	Sweden	1	​	​	1	​	​	​	​	​
	Other European countries	1.11	0.76–1.62	0.575	1.01	0.68–1.50	0.966	​	​	​
	Non-European countries	0.99	0.58–1.69	0.984	1.17	0.66–2.06	0.589	​	​	​
	Clinical experience									
	<5 years	1	​	​	1	​	​	​	​	​
	5–10 years	1.76	1.16–2.66	0.007	1.59	0.96–2.61	0.070	​	​	​
	10–15 years	2.11	1.38–3.24	<0.001	2.08	1.16–3.72	0.014	​	​	​
	>15 years	1.37	0.96–1.96	0.081	1.96	1.03–3.73	0.040	​	​	​
Positive attitudes to telemedicineconsultation	Age									
	≤29 years	1	​	​	1	​	​	1	​	​
	30–39 years	1.03	0.52–2.03	0.942	1.81	0.77–4.24	0.174	1.95	0.80–4.78	0.143
	40–49 years	0.79	0.40–1.55	0.487	1.87	0.73–4.82	0.195	2.04	0.76–5.49	0.159
	50–59 years	0.94	0.46–1.94	0.868	2.55	0.89–7.33	0.083	3.16	1.05–9.56	0.042
	≥60 years	1.18	0.57–2.47	0.653	3.74	1.23–11.38	0.020	4.65	1.45–14.95	0.010
	Gender									
	Male	1	​	​	1	​	​	1	​	​
	Female	1.16	0.86–1.57	0.344	1.31	0.95–1.81	0.097	1.35	0.96–1.90	0.083
	Employer									
	Region	1	​	​	1	​	​	1	​	​
	Private sector	1.38	0.99–1.92	0.054	1.41	1.00–1.99	0.050	1.07	0.74–1.56	0.705
	Other	1.59	0.70–3.59	0.265	1.58	0.68–3.71	0.291	1.32	0.54–3.20	0.541
	Country of education									
	Sweden	1	​	​	1	​	​	1	​	​
	Other European countries	1.21	0.79–1.84	0.378	1.48	0.95–2.31	0.084	1.51	0.94–2.42	0.086
	Non-European countries	2.09	1.03–4.23	0.041	2.13	1.01–4.49	0.047	2.12	0.98–4.58	0.057
	Clinical experience									
	<5 years	1	​	​	1	​	​	1	​	​
	5–10 years	0.68	0.41–1.14	0.142	0.49	0.26–0.94	0.031	0.45	0.23–0.88	0.019
	10–15 years	0.59	0.35–0.97	0.038	0.38	0.19–0.77	0.007	0.29	0.14–0.61	0.001
	>15 years	0.65	0.41–1.03	0.067	0.33	0.15–0.72	0.006	0.25	0.11–0.57	<.001

OR, odds ratio; CI, confidence interval.

^a^Crude.

^b^Multivariable adjusted for age, gender, employer, country of education, clinical experience, health, turnover intention and job satisfaction.

^c^Multivariable adjusted for age, gender, employer, country of education, clinical experience, health, turnover intention, job satisfaction and use of telemedicine.

**Table 3.** Logistic regressions for the associations between the use of and positive attitudes toward telemedicine consultations and self-rated general health, turnover intention and job satisfaction.

​	​	Model I			Model II			Model III		
		OR^a^	95% CI	*P* value	OR^b^	95% CI	*P* value	OR^c^	95% CI	*P* value
Use of telemedicine consultation	Health									
	Bad	1	​	​	1	​	​	​	​	​
	Good	1.25	0.91–1.71	0.168	1.19	0.84–1.68	0.337	​	​	​
	Turnover intention									
	High	1	​	​	1	​	​	​	​	​
	Low	0.76	0.53–1.10	0.151	0.60	0.38–0.94	0.025	​	​	​
	None	0.62	0.42–0.91	0.015	0.51	0.31–0.86	0.010	​	​	​
	Job satisfaction									
	Low	1	​	​	1	​	​	​	​	​
	Middle	1.37	0.88–2.15	0.166	1.67	1.01–2.76	0.045	​	​	​
	High	1.25	0.88–1.77	0.219	1.86	1.17–2.97	0.009	​	​	​
Positive attitudes to telemedicine consultation	Health									
	Bad	1	​	​	1	​	​	1	​	​
	Good	0.80	0.55–1.17	0.252	0.71	0.47–1.08	0.105	0.74	0.48–1.15	0.177
	Turnover intention									
	High	1	​	​	1	​	​	1	​	​
	Low	0.94	0.64–1.40	0.772	0.59	0.35–0.99	0.043	0.63	0.36–1.09	0.096
	None	0.90	0.59–1.38	0.629	0.52	0.29–0.93	0.027	0.58	0.31–1.07	0.082
	Job satisfaction									
	Low	1	​	​	1	​	​	1	​	​
	Medium	1.55	0.92–2.60	0.097	2.18	1.20–3.97	0.011	2.27	1.20–4.28	0.012
	High	1.62	1.07–2.46	0.023	3.03	1.70–5.40	<.001	3.21	1.74–5.90	<0.001
	Frequency of use of telemedicine									
	On some occasions	1	​	​	​	​	​	1	​	​
	On many occasions	3.82	2.75–5.30	<0.001	​	​	​	4.25	2.98–6.04	<0.001
	For almost all my patient consultations	8.12	0.99–66.63	0.051	​	​	​	8.02	0.93–69.14	0.058

OR, odds ratio; CI, confidence interval.

^a^Crude.

^b^Multivariable adjusted for age, gender, employer, country of education, clinical experience, health, turnover intention and job satisfaction.

^c^Multivariable adjusted for age, gender, employer, country of education, clinical experience, health, turnover intention, job satisfaction and use of telemedicine.

The study procedures and data collection were performed in accordance with the Helsinki Declaration.^37^ Respondents were informed in the invitation letter that participation was voluntary and confidential, and that they had the right to withdraw participation at any time. The Swedish Ethical Review Authority approved this study (Dnr: 2022-03275-01; 2022-00310-02).

## Results

### Characteristics of the study sample

Of the 1099 respondents, 36.7% were <40 years old and 18.1% were ≥60 years; 58.2% were women; 70.5% were employed in a region (public employer); and 79.1% received their medical education in Sweden. Four in ten respondents (40.3%) had 15 years or more of clinical experience.

Of all the respondents in the study sample, 70.5% had used telemedicine; 40.3% had used it on some occasions. 29.2% on many occasions and 1.0% for almost all patient consultations (Table 1).

A total of 94% (721 out of 768, Figure 1) of telemedicine users responded to the question on their attitudes towards telemedicine. Attitudes toward telemedicine use among respondents who answered the question were generally positive; 59.8% of users reported positive attitudes (431 out of 721 users, Table 1), and 40.2% expressed negative attitudes (290 out of 721 users, Table 1). Among those who reported using telemedicine on many occasions or almost always, just over half of the responders reported positive attitudes (56.6%, Table 1). At the same time, the situation was reversed for those who rarely used telemedicine, were 74.8% reported negative attitudes.

### Characteristics of primary care physicians’ self-reported use of and positive attitudes towards telemedicine consultations

Respondents aged 30–59 years were approximately twice as likely as physicians aged ≤29 years to report using telemedicine (Table 2), whereas those aged >60 did not differ significantly from the youngest group. Physicians employed in privately governed clinics were 2.5 times more likely to also report using telemedicine than respondents employed by a region. Longer clinical experience was associated with greater use of telemedicine. Physicians with 5–10 years of clinical experience were 1.76 times more likely, and those with 10–15 years of clinical experience were 2.11 times more likely to report using telemedicine than those with <5 years of clinical experience. No differences were observed between genders or countries of education. In the adjusted Model, age was no longer significant, whereas clinical experience remained significant. Other variables remained similar in the adjusted Model II.

Turning to self-reported attitudes toward telemedicine consultations, statistically significant differences were observed for physicians educated in non-European countries, who were more than twice as likely as those educated in Sweden to report positive attitudes toward telemedicine (Models I and II, Table 2). Clinical experience of 10–15 years was associated with a lower likelihood of reporting positive attitudes toward telemedicine compared with <5 years of clinical experience. The adjusted Model II showed some differences compared to the crude Model I. The oldest group was 3.74 times more likely to report positive attitudes compared to the youngest group. Privately employed physicians were 1.41 times more likely than physicians employed in regions to report positive attitudes toward telemedicine. Furthermore, in contrast to the crude analysis, age became a statistically significant factor.

In Model III, we additionally adjusted for telemedicine use, and the results showed that clinical experience remained statistically significant, as did older age. At the same time, the differences between the private and public sectors became nonsignificant.

### Associations with self-rated general health, turnover intention and job satisfaction

Table 3 shows the associations between the reported use of and positive attitudes toward telemedicine consultations and self-rated general health, turnover intention and job satisfaction. Regarding telemedicine use, no significant crude associations were observed, except for a reduced likelihood of reporting a telemedicine consultation among those who reported no turnover intention compared to higher turnover intentions. In the adjusted Model II (Table 3), both those who reported low and no turnover intention had a statistically significantly reduced likelihood of reporting telemedicine use, indicating an association between frequent telemedicine use and turnover intention. Furthermore, in Model II, job satisfaction became statistically significant. The results indicated that physicians who reported higher job satisfaction were also more likely to report use of telemedicine consultations.

The pattern is similar for attitudes to telemedicine consultation. No crude association was found between self-rated general health and attitudes to telemedicine consultation (Model 1). In Model I, there was an association between reported high job satisfaction and positive attitudes toward telemedicine. However, in the adjusted Model II, both turnover intention and job satisfaction were statistically significantly associated with attitudes. Those who reported no or low turnover intention were less likely to also report positive attitudes toward telemedicine consultations than those with high turnover intention. Meanwhile, compared to those who report low job satisfaction, those reporting high job satisfaction had three times the odds of also reporting positive attitudes toward telemedicine, and the respective odds for medium job satisfaction were just over two.

When adjusting for the reported frequency of use of telemedicine consultations (Model III), turnover intention was no longer statistically significant, while no changes were observed for general health and job satisfaction. Those who reported using telemedicine on many occasions were 4.25 times more likely to also report a positive attitude.

## Discussion

This study investigated primary care physicians’ self-reported use of and attitudes toward telemedicine consultations with patients in Sweden, and whether use and attitudes were associated with general health, turnover intention and job satisfaction. A nationally representative sample of physicians employed in Swedish primary care was surveyed cross-sectionally. Seven out of ten primary care physicians reported using telemedicine during patient consultations on some or many occasions. Among these, approximately 60% reported a positive attitude toward using telemedicine. Our study found some demographic variation; telemedicine consultations were more common among primary care physicians employed in the private sector and those with longer clinical experience. Meanwhile, those with longer experience working as physicians tended to report negative attitudes toward telehealth consultations. We found no associations between the use of and positive attitudes toward telemedicine consultations and general health. Both the use of and positive attitudes toward telemedicine consultation were associated with high job satisfaction. In contrast, those with high turnover intention were more likely to also use telehealth and to have positive attitudes toward it than those with no turnover intention; however, this association was no longer statistically significant after adjusting for the frequency of telemedicine use (Table 3). While previous research has largely focused on patient experiences or clinical effectiveness of telemedicine,^16,18,20–22^ fewer studies have examined how telemedicine adoption relates to physicians’ work-related experiences within primary care systems. By simultaneously examining demographic variation and associations with workforce factors such as job satisfaction and turnover intention, this study extends existing literature beyond utilisation rates and attitudes alone. Furthermore, by shifting the analytical focus to physicians’ implementation experiences and their relationship to workforce-related factors within a national primary care context, we contribute to practical implications.

Although telemedicine has been utilised in primary care and various clinical specialities for some time,^2^ the COVID-19 pandemic has underscored its importance in health systems worldwide.^4–7^ Telemedicine consultations and other digital tools will likely play an increasingly important role in the provision of primary care and may become a necessity for the general population. It is essential to gain insights into physicians’ use of and attitudes toward telemedicine to optimise its utilisation. We found that telemedicine was fairly widely used among Swedish primary care physicians and that most users were quite positive.

While use of and attitudes toward telemedicine consultations were associated with higher job satisfaction, they were also associated with higher turnover intentions. This seemingly paradoxical finding suggests a more complex relationship between digital transformation and the psychosocial work environment. One possible interpretation is that those who embrace telemedicine feel more satisfied due to increased flexibility, autonomy, or perceived modernisation of healthcare delivery, as shown in earlier research.^12,38–40^ At the same time, this group may also have higher expectations of organisational adaptability and innovation. When these expectations are not met, frustration or disillusionment may contribute to thoughts of leaving the job. Another interpretation could be that those who are more open to change and innovation may also be more mobile in the labour market and more likely to seek new opportunities, a possibility worth further exploration. Confidence in using telemedicine may also serve as a gateway to alternative jobs, where high job satisfaction can increase awareness of career options and new opportunities. This duality highlights the importance of not viewing digitalisation as a straightforward solution to job satisfaction or retention issues. Further longitudinal and mixed-method research is needed to disentangle these mechanisms and understand the conditions under which telemedicine contributes to sustainable work satisfaction and retention in primary care.

Cross-sectional surveys cannot inherently establish causality. Therefore, we cannot determine whether those with positive attitudes to telemedicine use it more frequently or if increased usage fosters more favourable attitudes. Additionally, we cannot ascertain if their attitudes changed over time with usage. Nonetheless, these insights, in conjunction with existing literature, may prompt speculation. First, the rapid implementation of telemedicine during the COVID-19 pandemic limited many primary care physicians’ autonomy to make usage decisions based on their attitudes or willingness.^7,41,42^ Furthermore, Swedish primary care usually operates under specific regulations governing the use of telemedicine. Consequently, physicians’ attitudes to telemedicine may not influence their actual use of telemedicine. Although many social-cognitive theories in psychology posit that attitude formation precedes behaviour, e.g., the Theory of Reasoned Action^43^ and the Theory of Planned Behaviour,^44^ research has shown that people often behave first and form attitudes afterwards or that attitude formation and behaviour occur in an interwoven process.^45,46^ Thus, the process of forming attitudes and behaviour can be seen as circular rather than one-directional: attitudes about a behaviour (e.g. attitudes concerning the use of telemedicine) influence the decision to engage in this behaviour, the execution of which is then evaluated, which in turn has an impact on the attitudes to the behaviour.^47^ Thus, it seems plausible that using telemedicine for patient consultations enables physicians to recognise the important benefits of this approach, which generates positive attitudes toward telemedicine use. This interpretation is supported by our previous research, which has shown that Swedish primary care physicians identified numerous strengths with telemedicine consultations, including positive patient responses, decreased workload and increased work flexibility.^12^ These advantages may not be as apparent to non-users, making them less likely to develop positive attitudes. Further longitudinal studies are warranted to investigate the factors motivating physicians to adopt telemedicine.

We found some demographic differences in reported use of and attitudes towards telemedicine. These findings provide contextual insight into how professional background and employment setting may shape implementation experiences within primary care. We found that longer clinical experience was associated with higher use of telemedicine consultations in our study. However, longer clinical experience also increased the likelihood of expressing negative attitudes toward telemedicine. Thus, longer clinical experience does not appear to be associated with more positive attitudes; rather, the extent of telemedicine use, regardless of clinical experience, is the key factor. This may also be linked to poorer technical readiness or literacy among older or more senior physicians.^48^ The use of telemedicine consultations does not necessarily imply that physicians possess the necessary technical knowledge and skills to do so, and their attitude towards its use may therefore be lower.

We also found that privately employed physicians were more likely to have used telemedicine for patient consultations. The development of telemedicine in Sweden has been led by private companies, which have offered digital consultations with physicians for nearly 10 years. This development was facilitated by the introduction of a new patient law in 2015 (2014:821), which aimed to strengthen the patient’s position and participation in healthcare.^49^ This law enabled residents to register at any primary care centre of their choice nationwide. The 21 publicly funded regions in Sweden are increasingly offering digital care, a development that was accelerated by the COVID-19 pandemic.^2^ Before the pandemic, there was considerable debate in Sweden concerning the pros and cons of using telemedicine in healthcare. Many physicians offered harsh criticisms of this way of working in the profession’s leading journal in Sweden (*Läkartidningen*). However, the debate over digital patient consultations subsided when the pandemic hit in 2020, prompting a rapid reorientation of healthcare operations and services, including an increase in telemedicine use. As new digital tools and AI are rapidly introduced in healthcare, it is important to follow usage and attitudes toward these tools.

### Implications

Despite the study’s cross-sectional design, the findings provide contextual insight into how telemedicine is currently used and perceived within Swedish primary care. The use of a national representative sample offers a broad view of implementation experiences across settings, although results should be interpreted in light of self-reported data and survey response patterns.

The present findings contribute to understanding the feasibility and workforce dimensions of telemedicine adoption. The observed variation in use and attitudes across levels of clinical experience highlights that telemedicine implementation may be experienced differently within the workforce. This suggests that digital transformation in primary care is not a uniform process but one shaped by professional background, work context, and organisational conditions. Implementing new digital tools can generate both benefits and challenges for the workforce. This underscores the importance of organisational support structures, such as technical infrastructure, clinical guidance, and opportunities for professional dialogue, when integrating digital consultations into routine care.

Moreover, health systems should approach digitalisation not merely as a technological upgrade but as a broader organisational change process that requires sustained engagement with clinicians. The impacts of telemedicine should therefore be systematically assessed to identify benefits for healthcare organisations, the workforce, and patients alike. Attending to these considerations may help maximise the advantages of telemedicine while mitigating unintended consequences for workforce stability.

### Strengths and limitations

This study has some limitations that must be considered when interpreting the findings. We employed a cross-sectional survey, which enables us to reach a large number of individuals in a short time; however, it does not permit causal inferences.^50^ The response rate was relatively low (34%), and we were unable to analyse the reasons for non-participation. As in much survey research, there is a risk that participants are those most interested in the topic or most capable of responding. Research has established that more motivated and opinionated individuals are more likely to respond to surveys.^51^ Additionally, factors such as a lack of motivation, high workload, poor timing, and inaccurate addresses may contribute to non-participation.^52^ Moreover, the LOHHCS data collection occurred during the last wave of the COVID-19 pandemic, when many physicians were under high strain, which may have prevented them from responding. Against this backdrop, it is difficult to determine whether those who responded to the survey held more favourable or more unfavourable attitudes toward telemedicine than the non-participants. If respondents were either more engaged with or more critical of telemedicine than non-respondents, the reported estimates of attitudes and experiences may not accurately reflect those of the broader physician population. As a result, the findings should be interpreted with caution, particularly when generalising to physicians who are less digitally engaged or more constrained by clinical workload.

Statistics Sweden distributed the questionnaires and provided the researchers with a technical report, including a dropout analysis for the full sample. To ensure the representativeness of the sample, Statistics Sweden compared the responses against the full sample and the entire population, utilising Swedish population registers to identify any systematic differences between these groups. This analysis revealed that the survey respondents were relatively more likely to be physicians born in the Nordic countries or with a Swedish background. No other characteristics showed systematic differences, such as gender, income, age or place of residence.

We lack precise information on the number of primary care physicians in the total physician population. Therefore, we do not know the precise response rate for this specific group. However, there is no indication that physicians in primary care would be either more or less likely to complete the questionnaire than those in other settings.

The above-mentioned limitations suggest that the generalizability of the findings is somewhat restricted and that further studies on the topic are warranted. In particular, the relatively low response rate raises the possibility of non-response bias, which may affect the extent to which the results can be generalised to the broader physician population. Although the dropout analysis conducted by Statistics Sweden did not reveal systematic differences between respondents and non-respondents with respect to key sociodemographic characteristics such as gender, age, income, or place of residence, it remains possible that unobserved factors, such as attitudes toward telemedicine, digital competence, or workload-related constraints, may differ between those who chose to participate and those who did not. Future studies employing higher response rates, longitudinal designs, or complementary qualitative approaches could help to further validate and contextualise these findings.

Further, single-item measures have both strengths and limitations. A strength of single-item measures is that they are brief, easy to administer, and reduce respondent burden, thereby enhancing response rates and data quality. The single items used in this study (general health, turnover intention, and job satisfaction) have been validated and are widely applied in occupational and health research, supporting their reliability and construct validity. However, a limitation of single-item measures is that they may capture a narrower aspect of a construct and provide less nuance than multi-item scales, thereby reducing sensitivity to subtle variations or multidimensional facets of the concept.

## Conclusions

In this cross-sectional national survey of Swedish primary care physicians, approximately seven in ten reported using telemedicine for patient consultations, and among users, around six in ten expressed positive attitudes toward its use. Physicians with longer clinical experience were more likely to use telemedicine but less likely to report positive attitudes toward it. The use of and positive attitudes toward telemedicine were associated with higher job satisfaction, but also with higher turnover intention. This pattern highlights the complexity of digitalisation in primary care and suggests that telemedicine adoption should not be viewed as a straightforward solution to workforce retention challenges.

Given the cross-sectional design and reliance on self-reported data, no conclusions can be drawn regarding the direction of the observed associations. The association between attitudes to and use of telemedicine may reflect reciprocal influences or shared underlying factors. These findings should therefore be interpreted as descriptive and exploratory, providing insight into how telemedicine is currently experienced within Swedish primary care rather than evidence of its impact on professional outcomes.

## Data Availability

The questionnaire data used in this study are restricted by the Swedish Ethical Review Authority to protect patient privacy. The respondents have consented to participate in studies conducted by this research group, but we do not have their consent to share the data.*
